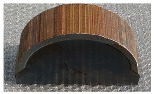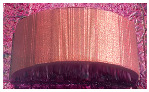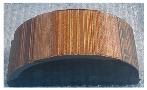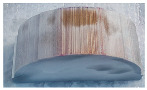# Residual Stress Reduction Technology in Heterogeneous Metal Additive Manufacturing

**DOI:** 10.3390/ma13235516

**Published:** 2020-12-03

**Authors:** Myoung-Pyo Hong, Young-Suk Kim

**Affiliations:** 1 Mechanical components and Materials R&D Group, Korea Institute of Industrial Technology, Daegu 711-883, Korea; mp77@kitech.re.kr; 2 Graduate School, Kyungpook National University, Daegu 41566, Korea; 3 School of Mechanical Engineering, Kyungpook National University, Daegu 41566, Korea

**Keywords:** additive manufacturing, residual stress, mold steel, functional metal powder, direct energy deposition

## Abstract

Metal additive manufacturing (AM) is a low-cost, high-efficiency functional mold manufacturing technology. However, when the functional section of the mold or part is not a partial area, and large-area additive processing of high-hardness metal is required, cracks occur frequently in AM and substrate materials owing to thermal stress and the accumulation of residual stresses. Hence, research on residual stress reduction technologies is required. In this study, we investigated the effect of reducing residual stress due to thermal deviation reduction using a real-time heating device as well as changes in laser power in the AM process for both high-hardness cold and hot work mold steel. The residual stress was measured using an X-ray stress diffraction device before and after AM. Compared to the AM processing conditions at room temperature (25 °C), residual stress decreased by 57% when the thermal deviation was reduced. The microstructures and mechanical properties of AM specimens manufactured under room-temperature and real-time preheating and heating conditions were analyzed using an optical microscope. Qualitative evaluation of the effect of reducing residual stress, which was quantitatively verified in a small specimen, confirmed that the residual stress decreased for a large-area curved specimen in which concentrated stress was generated during AM processing.

## 1. Introduction 

Recently, additive manufacturing (AM) processing technology has been gaining increasing interest globally. Additive processing technology refers to a molding technology that converts virtual 3D data into a tangible physical model. In particular, metal AM processing technology can be applied in high-value-added industries, such as aviation, aerospace, medical care, power generation, and automobile industries; therefore, studies on commercializing this technology are being actively conducted [1,2]. In ASTM F2792-12a, terms previously expressed in rapid prototyping technology were standardized as 3D printing or AM [3,4,5]. 

AM technology is classified according to the additive method as material extrusion (ME), material jetting (MJ), binder jetting (BJ), sheet lamination (SL), vat photo polymerization, powder bed fusion (PBF), and direct energy deposition (DED) [4,5]. Among these methods, metal AM uses the PBF and DED methods. The ME, MJ, BJ, and SL methods can also stack metals; however, they are not actively used. In the metal AM technology, the material cost is high, and the manufacturing time is long; therefore, research and application in relatively high-value-added industries are being actively conducted. 

Wilson et al. studied the remanufacturing technology of turbine blades using DED [6]. Shamsaei et al., Miedzinski, and DebRoy et al. studied the mechanical properties and reliability/durability of engineering parts based on the AM characteristics, thereby presenting a method for improving the mechanical properties of the output based on the AM process conditions [7,8,9]. Milewski and Yang et al. studied the applicability and mechanical properties of the products after AM in rocket nozzles, medical implants, and custom jewelry industries using the metal AM technology [1,2]. Ahn and Lee et al. studied the manufacturing process of the metal AM technology and its sustainable application in the field of eco-friendly manufacturing [10,11,12,13]. To improve the cooling characteristics in injection molding, Kim et al. studied a method of manufacturing injection molds with the same three-dimensional cooling channel as the product shape using a hybrid mold manufacturing technology that combines machining and AM processes, and they applied it to the injection molding of automotive switch cover products [14]. Park et al. studied the microstructure and heat treatment properties of mold steel materials using DED and the microstructure changes and energy input properties of materials during AM, and they suggested a method for improving the surface hardness properties of these materials [15,16]. Hong conducted a study on the shear mold manufacturing technology using the additive processing technology of dissimilar materials and introduced the case of commercialization [17]. Despite the very high metal powder material and process costs, metal additive processing has been recognized for its many advantages and is being actively commercialized. However, the deformation and residual stress that occur during the large-area AM of high-hardness metals remain a major problem that hinders the widespread application of this technology in the manufacturing industry. 

To solve the deformation and residual stress problems that occur during the AM process, previous studies have attempted to predict the thermal and mechanical behavioral effects of metal AM using finite element (FE)-based thermodynamic modeling and determine the optimal process parameters for the relaxation of the generated deformation and residual stress [18,19,20,21]. These previous studies investigated the AM process in a simple trial product unit for the thermodynamic analysis of the AM process, and only a few studies on the residual stress reduction technology considered the additive processing of large high-hardness materials. 

In this paper, a method for reducing the generation of residual stress that occurs during the large-area AM of high-hardness metal materials using DED is proposed. In DED, the powder is melted in a melting pool and rapidly solidified into a substrate or previously deposited layer repeatedly to create the desired shape. However, uneven cooling in the process of heating the partial part with a moving heat source during the AM process causes a thermal gradient, which creates residual stresses and strains, thereby reducing the mechanical strength and geometric accuracy of the manufactured product. Excessive residual stress generated during the DED is expected to cause cracks in the output. In this study, the amount of residual stress generated by changes in the laser power and the real-time base material-heating effect—factors expected to have the most influence on the generation of residual stress in the metal AM process—were evaluated for one type of cold work mold steel and one type of hot work mold steel (Fe-based materials). The residual stresses were measured using an X-ray stress diffraction device before and after AM. In addition, the mechanical properties of the specimens manufactured under each condition, based on their microstructure, were analyzed using an optical microscope. Finally, to confirm that the residual stress reduction effect, which was quantitatively verified in the small specimen unit, was similarly produced in the large-area specimen, the curved specimens in which concentrated stress occurred during the AM process were examined. The results were used to suggest a plan for reducing residual stresses.

## 2. Materials and Methods

Among the various DED equipment available, the AM equipment used in this study is the MX-311 (Insstek, Daejeon, Korea). This equipment can manufacture metal products identical to 3D CAD modeling by melting and laminating the base material using a 2 kW fiber laser and adding a spherical metal powder to the melting pool. Unlike PBF-type selective laser melting equipment, which is classified as soft-tooling equipment, this equipment uses a complete melting bonding method during additive processing. Hence, the mechanical properties of the finished output are equal to or better than that of bulk materials depending on the material, and it is therefore classified as hard-tooling equipment. Table 1 lists the characteristics of the soft and hard-tooling equipment. While the types of metal powders that can be used in most existing AM equipment is limited, the equipment used in this study allows for the use of various commercial metal powders, thereby making it AM equipment that is suitable for developing new metal powders with high material hardness and for researching commercialization technologies.

**Figure 1:**
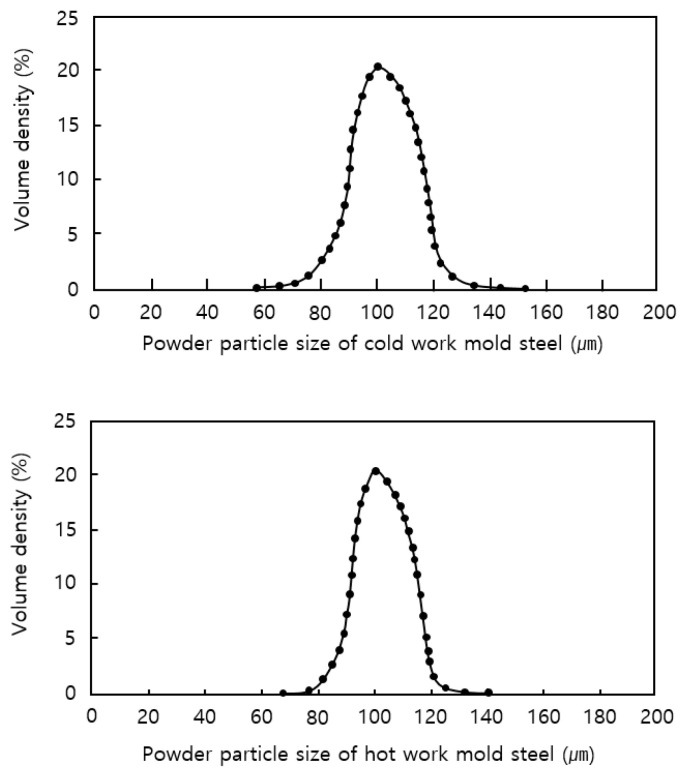
Metal powder particle size for laser deposition.

Table 2 lists the main alloy components of the substrate, cold work mold steel, and hot work mold steel materials employed in the experiment. The materials selected for the experiment are functional materials that can improve the cooling performance and manufacturing process by partially applying the metal AM technology to the functional part when making molds compared to the existing mold steel materials. The cold work mold steel material can achieve a high-hardness level of HRC60 by optimizing the AM process without an additional heat treatment process; the hot work mold steel material improves thermal conductivity by more than 40% compared to the existing H13 material. The materials applied in the experiment were composed of spherical particles with an average particle size of 100 μm using the gas atomizing method. Figure 1 shows the measurement results of the metal powder particle size.

**Figure 2:**
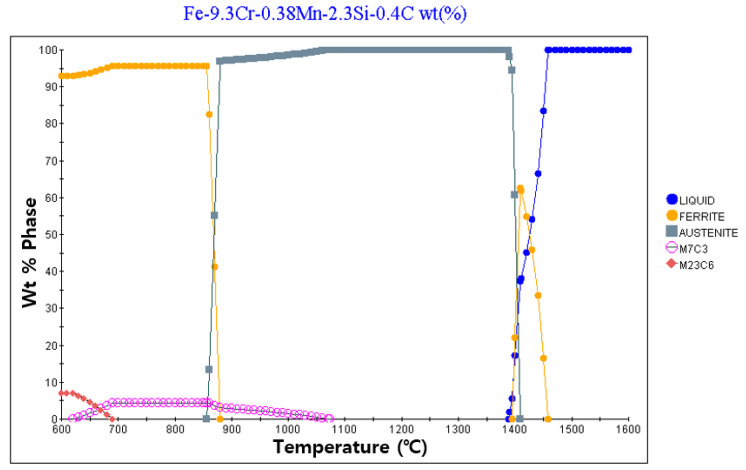
Equilibrium phase fraction of cold work mold steel for AM using JMatPro.

**Figure 3:**
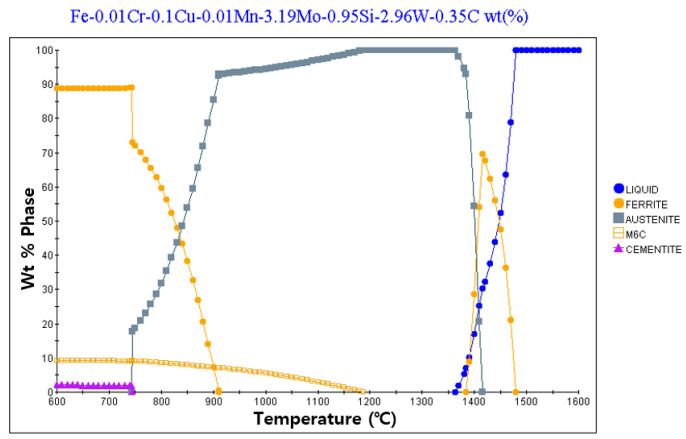
Equilibrium phase fraction of hot work mold steel for AM using JMatPro.

**Figure 4:**
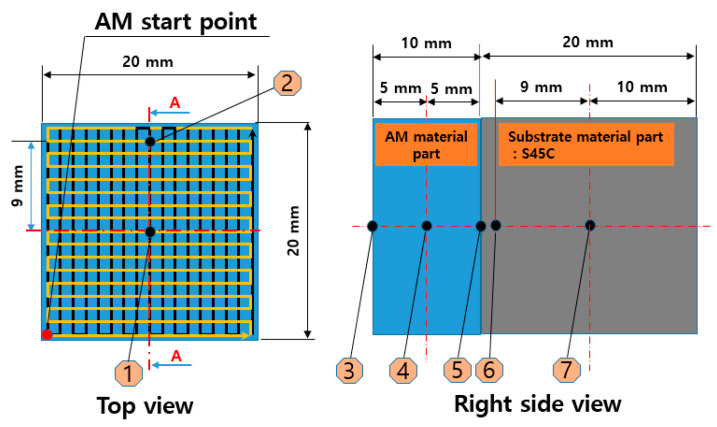
Concept used for fabricating a trial product to evaluate the residual stress according to the change in the AM process conditions and the location of the residual stress measurement.

Figure 2 and Figure 3 present the results of calculating the equilibrium phase fraction according to the temperature of the cold and hot work mold steel materials, respectively, using the JMatPro Version 7.0 software (Sentesoftware.co, Guildford, UK) based on the composition presented in Table 2. It can be observed that, based on the carbon content, austenite is formed, and carbide is crystallized from the liquid phase during solidification. In addition, ferrite is generated by precipitation at around 900 °C, and ferrite is seen to appear at around 1400 °C due to crystallization. However, it is predicted that, during actual solidification, the liquid phase section is extended owing to the non-equilibrium reaction, and a large amount of crystallized carbide is generated. Figure 4 depicts the trial product fabrication concept and the location of the residual stress measurement applied to the residual stress evaluation based on the change in the AM process conditions. The trial product was manufactured by performing AM at a height of 10 mm on an S45C base material (width = 20 mm, length = 20 m, and height = 20 mm). The residual stress was measured for two flat parts and five side parts after AM.

**Figure 5:**
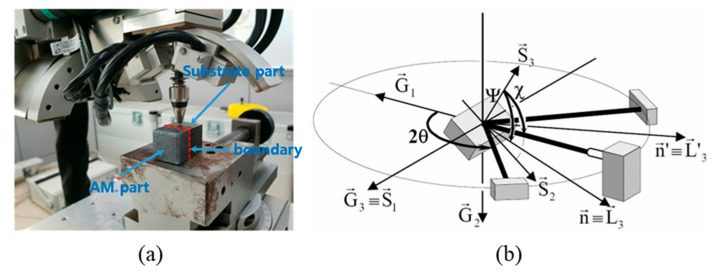
XRD residual stress measurement equipment and direction of residual stress analysis: (**a**) XRD residual stress measurement equipment; (**b**) direction of residual stress analysis.

Figure 5 presents the XRD residual stress measurement equipment (Xstress 3000, Stresstech Oy Co., Vaajakoski, Finland) and schematically the in-plane stress Ψ with respect to the two principal stress components σ_1_ (*S*_1_) and σ_2_ (*S*_2_). The basic principle of the XRD residual stress measurement device follows Bragg’s Law, which is established between the interatomic spacing and angle of incidence in the crystal when X-rays enter the metal crystal. The residual stress state in the workpiece affected by AM was analyzed by X-ray diffraction using the sin^2^Ψ method [23]. Table 3 shows the main XRD residual stress measurement conditions and the maximum measurement error value. In this table, the maximum error means that the positive value is tension, while the negative value is compression.

Table 4 and Table 5 list the main AM processes of the cold and hot work mold steel materials employed in this study, respectively. The surface roughness of the trial product laminated via DED was 100–200 μm based on the *R*_a_ value; the residual stress was evaluated without additional machining after the trial product was manufactured. 

**Figure 6:**
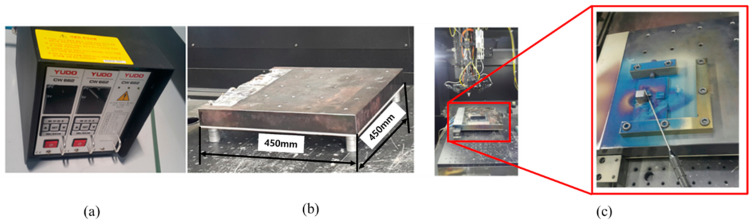
Real-time heating device using induction heater technology: (**a**) temperature controller; (**b**) heating table; (**c**) trial production example.

Figure 6 presents the real-time base material heating device based on the induction heater technology applied to the experiment. Induction heating refers to a method of heating a metal object using electromagnetic induction. When current is supplied to the coil, eddy currents are generated in the metal to be heated, and the Joule heating generated by the resistance of the metal raises the temperature. When the required temperature is set using the controller (Figure 6), the current flows through the coil in the heating table; simultaneously, the controller is used to compare the set temperature with the current temperature of the heating table in real time through the temperature measurement sensor built into the table. When heating the base material, the vacuum chamber is not applied, and the trial product is placed on a heating table in the air and heated to a specific temperature by heat conduction; further, the temperature is maintained to fabricate the trial product throughout the AM process. To avoid rapid cooling conditions when manufacturing a laminated trial product using the real-time heating technology, the entire base material is heated for a certain time until it reaches a specific set temperature, and the same temperature and holding time applied to the trial product manufacturing are maintained even after the trial product fabrication. Table 6 lists the holding time after reaching each set temperature after the trial product fabrication.

## 3. Results and Discussion

Table 7 shows the hardness and major thermal properties of the cold and hot work mold steel materials used in the experiment after AM. The thermal conductivity and thermal diffusion coefficient of hot work mold steel after AM were 32% and 34% higher than those of cold work mold steel, respectively. Compared with materials manufactured by conventional casting or forging techniques, materials manufactured by additive manufacturing have similar microstructures, there are, however, certain differences, such as the size and distribution of microstructures since they are manufactured by repeating local melting and cooling processes during manufacturing.

Table 8 lists the measurement results of residual stress based on the change in laser power during AM for the cold work mold steel metal powder material. The experiment shows the average values of the remaining three trial products excluding the minimum and maximum values after the fabrication of five trial products in each output condition. The amount of residual stress generated during AM did not change proportionally with the increase in laser power; further, no correlation was observed between the change in the laser power and the amount of residual stress generated during AM. In all measured trial products, the maximum residual stress occurred at point 4 (Figure 4), which is the midpoint of the laminated trial product.

Table 9 summarizes the amount of residual stress change during the production of trial products using AM under room temperature (25 °C) and real-time base metal heating conditions for the hot work mold steel material. The average values of the three remaining trial products, excluding the minimum and maximum values, after the fabrication of the five trial products under each temperature condition are evaluated. The residual stress was reduced by 35% at the base metal-heating temperature of 150 °C and by 57% at 250 °C when compared with the AM process condition at room temperature based on the evaluation of the point of maximum residual stress temperature condition. Further, in the AM process at room temperature, the maximum residual stress occurred at point 5 (Figure 4), which is at the interface between the substrate and AM materials; however, at 150 and 250 °C, the maximum residual stress occurred at point 4 (Figure 4). In addition, in Table 8 and Figure 4, Positions 1 and 2 in Figure 4 are the surface area manufactured by DED, while Position 7 is the center of the substrate material that is JIS_S45C. Considering the amount of stress change in the AM, it is expected that the magnitude of the stress on the surface portion that repeats melting and quenching by the AM will be larger than Position 7, which is a solid state. The tensile strength of the substrate base material is about 570 MPa, while the hot work mold steel material is approximately 1800 MPa. Therefore, as the temperature of the base material increases in the DED, the residual stress changes from compression to tension at the surface, while the residual stress at Position 7 showed that the tendency was different from tendency of other positions. It is decided to be because the tensile strength of the material manufactured by DED is about three times higher than that of the base material.

Table 10 and Table 11 summarize the results observed using an optical microscope (KH-7700; HiROX Co Ltd.; Tokyo, Japan) at the center of the trial product fabricated under the base material non-heating conditions of the hot work mold steel material and under the real-time base material heating conditions (250 °C), respectively. Each trial product was subjected to an etching process wherein a solution of hydrochloric acid + nitric acid + methanol (10 mL of HCl, 20 mL of HNO_3_, and 20 mL of CH_3_OH) and water (H_2_O) was diluted in a ratio of approximately 1:10 and applied to the product for 5 s. In the case of the trial product laminated under the non-heating condition, many carbides were observed, and serious corrosions were found around the carbides. Further, the carbide and general layers were clearly distinguished, which is attributed to the rapid cooling during or after AM. In contrast, in the case of trial products manufactured under real-time preheating and heating conditions (250 °C), the carbide and general layers were not distinguished, and the particles were small and uniformly distributed. This is attributed to a heat treatment effect caused by heating the base material during AM. 

**Figure 7:**
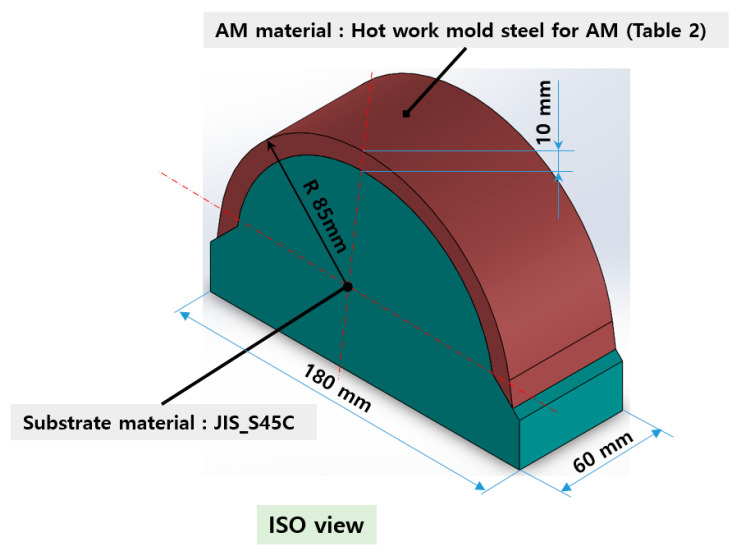
Large curved trial product shape and manufacturing concept.

The reduction in the residual stress caused by the application of real-time base material-heating technology during AM was evaluated quantitatively in a small trial product unit. The amount of residual stress generated under non-heated AM conditions and real-time base metal-heating conditions (250 °C) for large curved trial products, wherein concentrated stress occurred, was also evaluated qualitatively. Figure 7 shows the shape and manufacturing concept of the large curved trial product employed in the test. The JIS_S45C material was applied as the substrate material, and hot work mold steel (Table 2) was applied for AM. Table 12 presents the crack test result obtained using the liquid penetration test for large curved trial products manufactured under the non-heating condition. Owing to the excessive residual stress during AM, several cracks were observed, and the production of the trial product could not be completed. 

**Figure 8:**
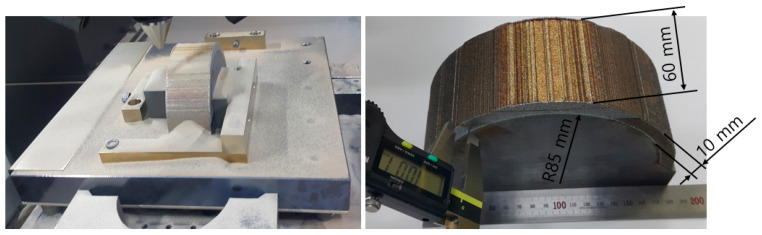
Large curved trial product manufactured using real-time base material-heating technology.

Figure 8 shows the same trial product manufactured under real-time heating conditions (250 °C). Unlike the non-heating AM condition, the residual stress reduction effect caused by the application of the real-time base material-heating technology enabled the production of the trial product without cracks occurring. Table 13 shows the crack test results obtained using the liquid penetration test for large curved trial products manufactured using the real-time base material-heating technology. The test results indicate that no cracks occurred; it is predicted that the residual stress decreased in the same manner as the evaluation result in the small trial product unit.

## 4. Conclusions

This study investigated a method for reducing residual stress during the AM process of Fe-based high-hardness hot work mold steel and cold work mold steel materials using heterogeneous metal AM technology. The following conclusions were drawn.

- The change in laser power during additive processing and the residual stress of the material exhibited no specific correlation. In the AM process, heating and cooling were repeated for a partial area; therefore, it is predicted that change in laser power did not significantly affect the change in temperature of the entire base material or the output.
- The reduction in residual stress is confirmed when applying the real-time heating technology in the sample-level experiment. In addition, the results of this study demonstrate that the maximum residual stress generation point can be changed by applying real-time heating technology.
- In the case of the hot mold work steel specimen manufactured under real-time heating conditions, as a result of observation with an optical microscope, the carbide layer and the general layer were not distinguished, and the particles were small and uniformly distributed. This is believed to be the result of the effect of reducing the temperature deviation during melting and cooling in the AM process.
- Moreover, reducing the residual stress was also confirmed on large-area curved specimens.

In future research, we intend to investigate crack generation and prediction technology using computer-aided engineering in order to reduce the residual stress that occurs during the AM of various metal products.

## Figures and Tables

**Table 1:** Characteristics of soft and hard-tooling metal additive manufacturing (AM) equipment [22].

Soft-Tooling Equipment		Hard-Tooling Equipment
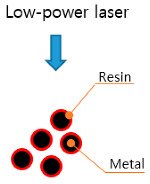	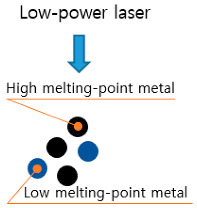	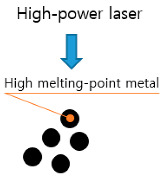
- Degreasing and sintering are required - Suitable for prototype production - Die fabrication possible using hot isostatic press equipment	- No restrictions of shape - PBF	- Degreasing and sintering are not required - Suitable for production of die and high-strength components - Restrictions of shape - DED

**Table 2:** Main alloy components (wt.%) of metal materials applied in the experiment.

Material Classification	C	Cr	Mn	Si	Ni	Cu	P	S	Mo	W	V	Fe
Substrate material	0.42	0.20	0.70	0.25	0.20	0.30	0.03	0.03	-	-	-	Balance
Cold work mold steel for AM	0.40	9.30	0.38	2.30	-	-	-	-	-	-	-	Balance
Hot work mold steel for AM	0.35	0.01	0.01	0.95	-	0.10	-	-	3.19	2.96	0.03	Balance

**Table 3:** XRD residual stress measurement conditions.

Parameter	Value
X-ray generator power (W)	30 KV.6.7 mA = 200
Radiation Cr K α (nm)	λ = 0.22911
2θ (°)	156.40
Miller indices (hkl)	[211]
Collimator size (mm)	φ 3
Detector pixel size	512
Arc radius (mm)	50
Exp. Time (s)	20
Tilts	5/5
Ψ angles (°)	−40/40
Ψ oscillation (°)	5
Young’s modulus (MPa)	211,000
Poisson ratio	0.3
Calculation cross correlation	Linear background
Measurement method	Modified X
Analysis method [23]	Sin^2^psi method
Maximum measurement error value (MPa)	±26.7

**Table 4:** Direct energy deposition (DED) conditions for trial products of cold work mold steel.

Parameter	Value
Supplied powder (g/min)	5.5
Supplied powder gas (l/min)	2.5
Supplied shield gas (l/min)	7.0
Laser power (W)	250–600
Deposition speed (m/min)	0.85
Deposition height per layer (mm)	0.25
Deposition width pitch per layer (mm)	0.5
Laser beam diameter (mm)	0.8
Laser wavelength (nm)	1060

**Table 5:** DED conditions for trial products of hot work mold steel.

Parameter	Value
Supplied powder (g/min)	6.5
Supplied powder gas (l/min)	2.0
Supplied shield gas (l/min)	7.0
Laser power (W)	300–450
Deposition speed (m/min)	0.85
Deposition height per layer (mm)	0.25
Deposition width pitch per layer (mm)	0.5
Laser beam diameter (mm)	0.8
Laser wavelength (nm)	1060

**Table 6:** Holding time according to temperature when fabricating the trial product using the heating table.

Set Temperature (°C)	Holding Time After Reaching the Set Temperature (min)
0–100	30
100–150	30
150–200	30
200–250	30

**Table 7:** Hardness and major thermal properties for each material after additive manufacturing.

Material Type	Measuring Temperature (°C)	Hardness (HRC)	Conductivity (W/mk)	Diffusivity (mm^2^/s)	*C*p (J/g/K)	Density (g/cm^3^)
Cold work mold steel	25	58–60	14.340	4.162	0.459	7.512
Hot work mold steel		50–52	21.107	6.378	0.419	7.898

**Table 8:** Residual stress generation according to the laser power change during AM of cold work mold steel material.

Measurement Location	Residual Stress Generation Amount According to Laser Power Change (MPa)			
	Variable Laser Power 250–600 W	Fixed Laser Power 350 W	Fixed Laser Power 450 W	Fixed Laser Power 550 W
1	262	268	265	285
2	232	240	271	263
3	171	238	175	200
4	305 *	483 *	329 *	301 *
5	−243	34	−228	−99
6	−212	−147	−130	−176
7	−61	102	−121	−41

* The point of occurrence of the maximum residual stress of the trial product under the laser power condition during AM.

**Table 9:** Residual stress generation according to the application of real-time base material-heating technology in the AM of hot work mold steel material.

Measurement Position	Residual Stress Generation Amount According to Base Material-Heating Temperature Change (MPa)		
	25 °C	150 °C	250 °C
1	−140	−80	83
2	−153	−103	128
3	278	179	132
4	495	480 *	317 *
5	747 *	378	78
6	506	279	−42
7	−63	52	−35

* Maximum residual stress of trial product under the base material heating temperature condition during AM.

**Table 10:** Laminated trial products of the hot work mold steel material at the base material non-heating conditions, observed with an optical microscope.

Whole Trial Product	Area “A” Enlarged View	Area “B” Enlarged View	Area “C” Enlarged View
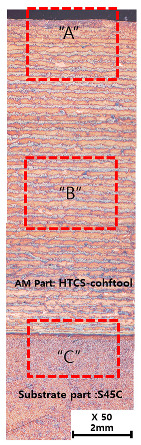	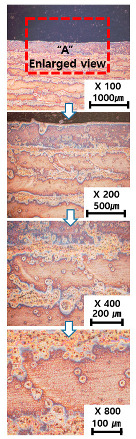	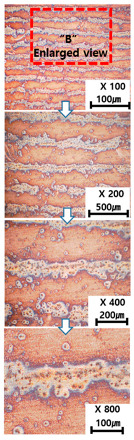	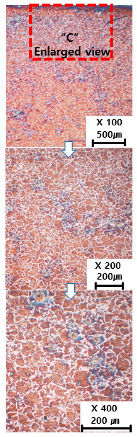

**Table 11:** AM trial products of the hot work mold steel material at 250 °C (real-time heating condition), observed with an optical microscope.

Whole Trial Product	Area “A” Enlarged View	Area “B” Enlarged View	Area “C” Enlarged View
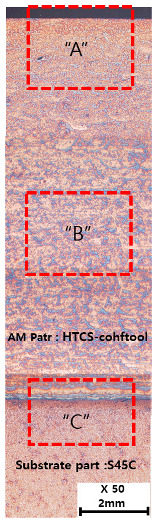	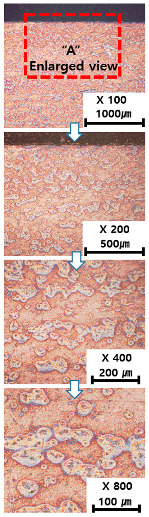	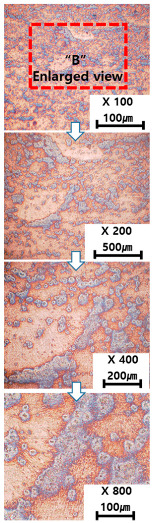	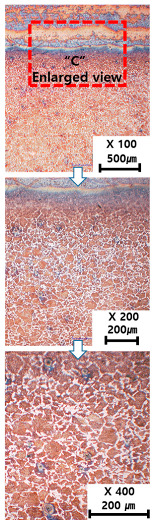

**Table 12:** Crack inspection using the liquid penetration test for large curved trial products fabricated under substrate material non-heating conditions.

First Trial Product Cleaning	Penetrant Application	Second Trial Product Cleaning	Crack Inspection
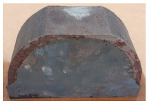	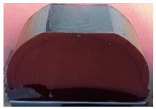	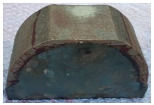	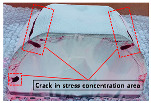

**Table 13:** Crack occurrence test results for large curved trial products using real-time substrate material-heating technology.

First Trial Product Cleaning	Penetrant Application	Second Trial Product Cleaning	Crack Inspection